# A Case of Cæsarian Section; Rendered Necessary by the Presence of a Tumor in the Vagina

**Published:** 1848-05

**Authors:** W. H. Byford

**Affiliations:** Mt. Vernon, Ind.


ART. II.—*A Case of Cæsarian Section; rendered necessary by the presence of a Tumor in the Vagina.* By W. H. BYFORD. M.D., of Mt. Vernon, Ind.

Mrs. Barlow, a farmer's wife, aged 26 years, was taken Nov. 13th, 1847, in labor with her third child. The midwife stated that she was called nine hours after labor commenced, which was at 7 o'clock P. M., on that day. At that time, the pains were hard, bearing down, and of considerable duration; she could not feel the membranes, but a large round body presented itself, which seemed to be about the size and feel of a child's head, of firm and hard consistence, just within the labia, admitting the fingers to pass up on the right side of the pelvis. She sat down by the bed side believing that a few more pains would deliver the head, and that the labor would soon be terminated. About 3 o'clock, A. M., on the 14th, while her fingers were in the vagina she felt a gush of water, and by passing the fingers high up on the right side of the pelvis she felt the scalp of what she supposed to be another child's head, which she thought solved the mystery of the detention, believing that two heads were engaged in the pelvis at the same time, one impeding the progress of the other. At 6 o'clock I was sent for, but being absent, the messenger returned without a physician; during the night he again came to town but with no better success, and it was not until 3 o'clock in the morning of the 16th that my partner, Doctor Conyngton, and myself visited her, when we found things as above stated. The waters had now been discharged over forty-eight hours,—pulse 110, face flushed, and the pains, which were very hard and frequent, had continued about the same, according to the statement of the midwives, of whom there were now two present, since 7 o'clock on the 13th, permitting the patient to take no rest. Upon examination per vaginam, a large osseous tumor was found springing from the plane and tuberosity of the ischium, nearly filling up the pelvis, leaving only one inch and a half space between its surface and the opposite ischiatic plane, and extending to within the same distance of the sacrum, allowing of more space in the region of the right ischiatic notch. The fingers could be made to pass up over the tumor to the linea ilio-pectinea, which might be felt, as also the sacro-iliac junctions. The mucous membrane of the vulva, when the labia were widely separated, was seen to form a continuous covering to the tumor as far as it could be traced by the eye. No amount of pressure compatible with the integrity of the soft parts made any impression on the tumor, or in the least altered its position. The head of the child was found at the superior strait, pressing firmly down upon the tumor with occiput to the symphisis pubis. Doctor Newman now joined us in consultation. The practicability of craniotomy was first discussed. But it seemed evident to us all that in consequence of the smallness of the opening through the pelvis. and the distance which the head was removed from the external outlet, we could not lessen the child sufficiently to bring it through—hence hysterotomy seemed the only possible means of relief; and even, without further loss of time, this afforded but small hopes of succes, from the unfavorable state of the patient.

The nature of the case was made known to the patient and her friends, and we were particular in stating the arguments for and against the operation. They readily consented, after proper consideration, to any measure we thought might afford hope of recovery or respite from her sufferings.

By the time the preparations within our reach were made it was dark, and I was compelled to operate by candle-light. The patient was placed near the edge of the bed, with her right side towards me. I took my position to her right, facing her head, so that I could have free use of my right hand. Being thus situated, I performed the operation, assisted by Doctors Conyngton and Newman, in the following manner: I made a free incision on a line with the right semilunaris, extending from an inch below a line drawn horizontally from the umbilicus, seven inches in extent, towards the pubis, dividing the integuments and adipose tissue. I then by several other strokes of the knife divided the different layers until I arrived at the peritoneum. After having exposed it for about two inches in length, in the upper angle of the wound, I raised a small portion by the forceps and divided it to a small extent, then cutting upon a grooved director until I could pass the fore-finger of the left hand into the wound, I directed the bistoury to the lower edge of the wound in the integuments. This being done the uterus was exposed to the extent of about six inches, the parietes of which were next divided by a few perpendicular strokes of the knife from above downwards, about an inch in extent, when the fore-finger again served as a director to the knife in conducting it to the lower extremity of the external wound. The membranes were now visible, and the shape of the child's limbs was seen through them. Lacerating these with the fingers, the child's feet, which lay in full view, were grasped, and the whole fœtus easily withdrawn.

I ought to have stated that we were satisfied of the death of the child prior to the commencement of the operation. It was above the medium size, and would have weighed nine pounds at least. Immediately after the removal of the child the hand was introduced into the wound, and the placenta and membranes taken away before the uterus had contracted much. It very soon assumed about the dimensions natural to it after delivery in ordinary cases. Pressure was kept up on each lip of the wound, from the time the external incision was completed, against the uterine parietes, in order to prevent the effused blood from entering the peritoneal sac. The object of the operation being thus accomplished, and the hemorrhage which was very inconsiderable, having ceased, the lips of the external wound were brought in complete apposition by means of four stitches and adhesive strips, except a small part of the lower angle which was now left open for the escape of any discharges formed in the wound itself. A compress over the wound and a roller around the abdomen completed the dressing, the whole operation having occupied about fifteen minutes.

The condition of the patient seemed to be very little altered, the pulse continuing about the same in frequency as before the operation. The tumor was again examined, and presented the same character as before.

We prescribed pulv. Dov. grs. viii. sulph. morph. gr. ¼, every four hours; patient to take no nourishment but bread and water.

17th, 8 o'clock, A. M., twelve hours after the operation. Patient slept some during the night; rested without pain until towards morning; pulse 120 rather hard and full. Abdomen a little swollen and tender, particularly on the left side; complains of oppression at the heart, and of pain, though slight, all over the left side and lower part of the abdomen. Bled to ℥xvi from the arm, and directed the abdomen to be fomented over the seat of the pain and soreness; continue the anodyne. The venesection relieved the precordial oppression, pain and tenderness to an extent that extorted from our patient expressions of gratitude; the pulse fell to 110, and grew more soft.

*Six o'clock, P. M.*, swelling, pain, tenderness, and oppression have returned, with the same frequency of pulse, which is decidedly hard. Bled to ℥ xx. with marked improvement of all the symptoms; ordered to continue medicines and fomentations.

18th, 8 o'clock, A. M. To all appearance the patient is better; has had a tolerable night's sleep, excepting a short time in which she was troubled by nausea and slight vomiting; pulse 110, but soft. Skin moist; has urinated four times since the operation without difficulty; some coagulated blood passed from the vagina through the night, with ordinary after pains; has had no alvine discharge since the operation. Directed her to discontinue the opiate, and take calomel grs. xx, to be followed in four hours with senna and sulph. magnesia.

*Five o'clock.* The purgative has operated three times copiously, bringing away dark colored stools. The patient is much worse; her pulse 140 and feeble; surface bathed in cold clammy perspiration,—extremities cold, vomiting, great listlessness and apathy, countenance pale, great oppression in hreathing—in short, sinking very rapidly. She expired at 8 o'clock next morning.

During the last twenty-four hours her abdomen was not swollen, tender nor painful, but the peculiar oppression about the heart continued until death.

The patient had a node on the left tibia, and the superior third of the right tibia was irregular along the surface.

The first labor of this woman was not more than ordinarily severe, tedious or difficult, but the second, which occurred on the 28th of August, 1845, was extremely so, and ever since that time she has complained of pain in the left side of the pubis. No autopsy was made.

It may be proper in conclusion, to state my reason for operating in the linea semilunaris instead of the linea alba, as is the general practice. It was because the uterus, in consequence of its right oblique position, offered its side to the knife in the last named place.

March 1848.

